# Distinct transcriptional and metabolic profiles associated with empathy in Buddhist priests: a pilot study

**DOI:** 10.1186/s40246-017-0117-3

**Published:** 2017-09-02

**Authors:** Junji Ohnishi, Satoshi Ayuzawa, Seiji Nakamura, Shigeko Sakamoto, Miyo Hori, Tomoko Sasaoka, Eriko Takimoto-Ohnishi, Masakazu Tanatsugu, Kazuo Murakami

**Affiliations:** 1 Department of Food and Nutrition, Tokyo Kasei University, Itabashi, Tokyo, Japan; 2 Foundation for Advancement of International Science, Kasuga, Tsukuba, Japan; 3 Division of Health Sciences, Tsukuba University of Technology, Kasuga, Tsukuba, Japan; 4 Departmemt of Neurosugery, Center for Integrative Medicine, Kasuga, Tsukuba, Japan; 5 DNA Chip Research Inc., Minato, Tokyo, Japan; 6 National Center for Child Health and Development, Tokyo, Japan; 7 Graduate School of Medicine, Kyoto Prefectural University of Medicine, Kyoto, Japan; 8 The Institute of Esoteric Culture, Koyasan University, Ina, Wakayama Japan

## Abstract

**Background:**

Growing evidence suggests that spiritual/religious involvement may have beneficial effects on both psychological and physical functions. However, the biological basis for this relationship remains unclear. This study explored the role of spiritual/religious involvement across a wide range of biological markers, including transcripts and metabolites, associated with the psychological aspects of empathy in Buddhist priests.

**Methods:**

Ten professional Buddhist priests and 10 age-matched non-priest controls were recruited. The participants provided peripheral blood samples for the analysis of gene expression and metabolic profiles. The participants also completed validated questionnaires measuring empathy, the Health-Promoting Lifestyle Profile-II (HPLP-II), and a brief-type self-administered diet history questionnaire (BDHQ).

**Results:**

The microarray analyses revealed that the distinct transcripts in the Buddhist priests included up-regulated genes related to type I interferon (IFN) innate anti-viral responses (i.e., MX1, RSAD2, IFIT1, IFIT3, IFI27, IFI44L, and HERC5), and the genes C17orf97 (ligand of arginyltranseferase 1; ATE1), hemoglobin γA (HBG1), keratin-associated protein (KRTAP10-12), and sialic acid Ig-like lectin 14 (SIGLEC14) were down-regulated at baseline. The metabolomics analysis revealed that the metabolites, including 3-aminoisobutylic acid (BAIBA), choline, several essential amino acids (e.g., methionine, phenylalanine), and amino acid derivatives (e.g., 2-aminoadipic acid, asymmetric dimethyl-arginine (ADMA), symmetric dimethyl-arginine (SMDA)), were elevated in the Buddhist priests. By contrast, there was no significant difference of healthy lifestyle behaviors and daily nutrient intakes between the priests and the controls in this study. With regard to the psychological aspects, the Buddhist priests showed significantly higher empathy compared with the control. Spearman’s rank correlation analysis showed that empathy aspects in the priests were significantly correlated with the certain transcripts and metabolites.

**Conclusions:**

We performed in vivo phenotyping using transcriptomics, metabolomics, and psychological analyses and found an association between empathy and the phenotype of Buddhist priests in this pilot study. The up-regulation of the anti-viral type I IFN responsive genes and distinct metabolites in the plasma may represent systemic biological adaptations with a unique signature underlying spiritual/religious practices for Buddhists.

**Electronic supplementary material:**

The online version of this article (10.1186/s40246-017-0117-3) contains supplementary material, which is available to authorized users.

## Introduction

Spirituality/religiosity is one of several unique aspects of human social environments and generally consists of psychological aspects accompanied by one’s behavior and social relationships. According to psycho-neuro-immune models of health regulation, spirituality/religiosity can be positively associated with psychological and physical health [1, 2]*.* Spirituality/religiosity provide coping resources that may improve mental health by increasing the frequency of positive psychological aspects and positive emotions [3, 4]. These positive psychological aspects include empathy and altruism, and the beneficial positive emotions include general well-being, happiness, and self-esteem [1, 5]. Coping resources also reduce negative stress that could result in emotional disorders, such as depression or anxiety. Because greater physical activity is associated with better mental health [6, 7], spirituality/religiosity should have a favorable impact on the physical state by balancing mental health. Furthermore, longitudinal studies by Miller et al. revealed that a high personal importance of spirituality/religiosity was associated with thicker cortices in certain brain regions (i.e., the left and right parietal and occipital regions, the mesial frontal lobe of the right hemisphere, and the cuneus and precuneus in the left hemisphere) and may confer protective benefits against the depressive symptoms in individuals with a high familial risk of major depression [8].

The surrounding social environments can change the basal expression profiles of certain genes (i.e., basal transcriptome) that are critical for the internal biological processes in our body [9, 10]. Notably, these effects are reliably associated with psychological states and are often induced by individuals’ subjective perceptions of their experiences in the surrounding social environments [11]. Stressors can activate the autonomic nervous system and/or the hypothalamic-pituitary-adrenal (HPA) axis to allow for physiological adaptation [12]. Individuals can manage these perceived stressors if the stressors fall within their coping abilities. However, once events or environmental demands exceed one’s coping ability, psychological stress ensues [11, 12]. Perceived adverse environmental events, such as psychological stress (e.g., perceived social isolation, bereavement, or long-term caregiving), could elicit negatively affected states, such as feelings of anxiety or depression. These negative psychological states, in turn, directly influence neural-endocrine processes with an immune dysfunction to enhance susceptibility to disease (e.g., viral infection, cardiovascular disease, and type II diabetes) and shape complex behavioral phenotypes (e.g., poorer sleep or appetite and addiction) [11, 12]. Meanwhile, these adversity-associated processes can influence the genome function. Consequently, these stressors can change the basal gene expression activity in peripheral blood leukocytes by up-regulating the expression of pro-inflammatory genes (e.g., interleukin-1β (IL1B), interleukin-8 (IL8), and tumor necrosis factor (TNF)) and down-regulating the expression of genes involved in type I interferon (IFN) innate anti-viral responses (e.g., IFIT-family genes and MX-family genes) [9, 10]. Due to the sensitivity of leukocytes to social adversity, these adverse experiences may shift the leukocyte’s basal transcriptional resources from a default anti-viral defense mode to an inflammation-promoting mode. In contrast, Fredrickson et al. have recently shown that a positive psychological state and social conditions (e.g., well-being) could oppose the adversity-associated transcriptome in peripheral blood [13, 14]. In another experimental design, we presented evidence that mirthful laughter with positive emotion can up-regulate distinct genes in the peripheral leukocytes of type II diabetes patients and adjust the activity of natural killer (NK) cells [15].

Notably, most studies reporting a positive connection between spirituality/religiosity and health have focused on “Christian” populations. These studies have typically been conducted in Western and Middle Eastern countries that are generally religious and economically well developed [2]. In contrast, a limited number of studies have been performed in non-Christian unique religious cultures in China [16] and Japan.

In this pilot study, we investigated the impacts of spirituality/religiosity on the functional activity of the human genome and metabolic reactions required for social regulation in qualified Buddhist priests. The objectives of this study were to (1) characterize the basal gene expression and metabolite profiles of qualified Japanese Buddhist priests; (2) evaluate the empathy psychological aspects in the priests, which are core elements of the fundamental concept of Buddhism [17, 18]; and (3) examine the association between empathy and the identified molecular markers.

## Methods

### Recruitment of participants

The data in this study were collected as a part of a research project that was conducted over 3 years to explore the role of spiritual/religious involvement across a wide range of biological markers. Ten male Japanese Buddhist priests and 10 male healthy age-matched Japanese non-priest controls were recruited from six different urban or rural areas (e.g., Tokyo, Kanagawa, Ibaraki, Kyoto, Gunma, and Wakayama in Japan) via leaflets and electronic media.

All Buddhist participants in this study belonged to the “Shingon” sect of Japanese Buddhism [19] and have performed the spiritual/religious main practices and duties for more than 7 years (median length, 15.50 years; range 7–35 years). These participants were qualified priests and commuted daily from their own homes to temples in towns and cities. All of the non-priest controls had steady full-time jobs and commuted daily from their own residences to their workplaces in towns and cities. Their occupational titles included manager, professional, technician, office worker, and service worker. Additionally, the non-priest controls had not received any training related to meditation or religious practices. The commuting methods of both the priests and the non-priest controls were similar and included travel on foot, using one’s own car or via public transportations, with commutes completed within approximately 50 min.

Both the priests and the non-priest controls met the following criteria: (i) decent health status with no subjective symptoms in the previous month and (ii) no history of receiving any dietary counseling or therapy from a doctor or dietitian. All participants were given a complete explanation of the study, which was approved on September 11, 2013, by the ethics committee of Tsukuba University of Technology (approval number TUT 20130911) and provided written informed consent. All participants were paid for their participation. During the study, the participants were asked a series of questions about their age, physical health, and medication use. The participants included a heterogeneous group of middle-aged adults (29–52 years old) as shown in Additional files 1, 2, 3 and 4. One priest had a previous history of venous thrombosis and had been administered an anticoagulant agent (Warfarin). He was also taking an antihypertensive drug (candesartan). Two priests suffered from diabetes; one of which was prescribed oral hypoglycemic agents (glimepiride and sitagliptin phosphate hydrate) and rosuvastatin to treat the accompanying hypercholesterolemia, and the other priest declared his diabetic nephropathy. In this pilot study, we did not exclude these three priests due to the limited number of recruited priests. A general serum chemical analysis and hematological tests were performed to examine their general conditions. These results are presented in Additional file 1. All participants had relative proportions of the peripheral blood cell types within normal ranges (27–90% neutrophils, 20–51% lymphocytes, 2–12% monocytes, 0–3% basophils, and 0–10% eosinophils).

### Measures

#### Genome-wide expression profiling

For RNA preparation, 2 × 2.5 mL samples of peripheral blood were drawn into PaxGene Blood RNA tubes (PreAnalytiX/QIAGEN Inc., Valencia, CA) from each subject. After blood collection, PaxGene tubes were left stand at room temperature (RT) for 2 h to ensure complete lyses of all blood cells, followed by stored at 4 °C for overnight. All tubes were stored at − 80 °C until RNA isolation. Based on the convenience of the participants, we performed blood sample collection on three different days (Dec 8, 2013, Apr 28, 2014, and Jun 8, 2014) within 6-month period. Total RNA was isolated within 6 months after storage. Total RNA was isolated using PaxGene Blood RNA Kit (PreAnalytiX/QIAGEN) according to the manufacturers’ instructions. Quantity and purity of the RNA was tested using the NanoDrop ND-1000 spectrophotometer (Thermo Fisher Scientific, Waltham, MA) and the Agilent 2100 Bioanalyzer (Agilent Technologies, Santa Clara, CA). In any event, only high-quality RNA samples containing intact 18S and 28S RNA were used for subsequent microarray and quantitative RT-PCR analyses.

#### Microarray analysis

One hundred nanograms of the total RNA was converted to complementary DNA (cDNA), amplified, and labeled with Cy3-labeled CTP using the Low Input Quick Amp Labeling Kit (Agilent Technologies, Santa Clara, CA) according to the protocol supplied by the manufacturer. Following labeling and clean up, the amplified RNA and dye incorporation were quantified using the NanoDrop ND-1000 spectrophotometer (Thermo Fisher Scientific, Waltham, MA). We used Agilent SurePrint G3 Human GE v2 8x60k Microarrays (Agilent Technologies, Santa Clara, CA) containing 50,599 unique genes. All samples were assayed in a single batch. After hybridization at 65 °C for 17 h, the arrays were washed consequently using the Gene Expression Wash Pack (Agilent Technologies, Santa Clara, CA). The microarrays were scanned using the Agilent Scanner, and the fluorescence intensities of the scanned images were quantified using Feature Extraction software ver. 10.7.3.1 (Agilent Technologies, Santa Clara, CA). Normalization was performed using Agilent GeneSpring GX version 13.1.1. (per chip normalization, 75 percentile shift; per gene normalization, none) with the range of expression intensities for inter-microarray. Only those genes whose expression data were available in more than 50% of hybridizations were included for further analysis [20]. Microarray raw data were deposited in the National Center for Biotechnology Information Gene Expression Omnibus (accession number GSE77676).

#### Differential expression analysis: the rank products method

Due to small sample sizes (*n* = 10 for each group) in this study, we could not evaluate whether transcriptional data showed the normal distribution. Therefore, we chose to employ the Rank Products Method, a “non-parametric” test as a cautious approach to determine significantly differential expressed genes. This method is reported to be relatively powerful especially for small sample sizes and when the data is non-homogeneous [21]. A gene considered as significantly differential expressed if the adjusted *P* value (false discovery rate, FDR) was equal to or less than 5% (0.05).

#### Quantitative RT-PCR analysis

We quantified the expression of 12 selected genes identified in the priests, including anti-viral signaling molecules (MX1, RSAD2, IFIT1, IFIT3, IFI27, IFI44L, HERC5), DEFA4, FOLR3, HBG1, C17orf97, and S100P through quantitative real-time reverse transcription polymerase chain reaction (qRT-PCR). cDNA was synthesized from 1000 ng total RNA using a High-Capacity cDNA Reverse Transcription Kit (Thermo Fisher Scientific). PCR reactions were carried out using ABI7500 Real Time PCR System (Thermo Fisher Scientific). Specific sets of primers and TaqMan probes were obtained from Thermo Fisher Scientific. Gene expression level of the target transcript was normalized with the values of an endogenous control gene (GAPDH). The data were normalized using the ∆*C*_*T*_ method (∆*C*_*T*_ = ∆*C*_*T*_ target––∆*C*_*T*_ control) by measuring cycle threshold ratios between candidate genes and an internal control gene, GAPDH. Expression level was described inversely as “− ∆*C*_*T*_”; thus, higher relative quantities indicate greater expression of target genes [20]. Differences of each transcript between the priests and the controls were compared using a Mann–Whitney *U* test, genes were considered as significantly differential expressed if the *P* value was equal to or less than 5% (0.05).

### Metabolomics analysis

Peripheral blood samples were collected in Vacutainer tubes voted with ethylendiaminetetraacetic acid (VP-NA070K; Terumo Corporation, Tokyo, Japan) and immediately centrifuged at × 1200*g* for 10 min to separated plasma. The plasma samples were then frozen in dry ice and stored at − 80 °C until use. The Human Metabolome Technologies, Inc., (Tsuruoka, Japan) performed all metabolome analysis [22].

For “hydrophilic” metabolites, capillary electrophoresis (CE)-coupled a time-of-flight mass spectroscopy (TOF/MS) was performed. Briefly, 50 μL of plasma samples were mixed with 450 μL methanol containing internal standards (solution ID H3304-1002, Human Metabolome Technologies, Inc., Tsuruoka, Japan) at 0 °C in order to inactivate enzymes. Then, 500 μL chloroform and 200 μL Milli-Q water were added, and the mixed solution was centrifuged at × 2300*g* for 5 min at 4 °C. The 350 μL of upper aqueous phase was filtered through a Millipore 5-kDa cutoff filter to remove proteins. The filtrate was centrifugally lyophilized and dissolved in 50 μL of Milli-Q water for following CE-TOF/MS analysis. CE-TOF/MAS was carried out using an Agilent CE Cappilary Electrophoresis System equipped with an Agilent 6210 Time of Flight mass spectrometer, Agilent 1100 isocratic HPLC pump, Agilent G1603A CE-MS adapter kit, and Agilent G1607A CE-ESI-MS sprayer kit (Agilent Technologies, Waldbronn, Germany). The systems were controlled by Agilent G2201AA ChemStation software version B.03.01 for CE (Agilent Technologies, Waldbronn, Germany). The metabolites were analyzed by using a fused silica capillary (50 μm *i.d.* × 80 cm total length), with commercial electrophoresis buffer (solution ID H3301-1001 for cation analysis and H3302-1021 for anion analysis, Human Metabolome Technologies) as the electrolyte. The sample was injected at a pressure of 50 mbar for 10 s (approximately 10 nL) in cation analysis and 25 s (approximately 25 nL) in anion analysis. The spectrometer was scanned from *m*/*z* 50 to 1000.

For “hydrophobic” metabolites, liquid chromatography (LC) time-of-flight mass spectrometry (TOFMS) was performed. Briefly, 500 μL of plasma samples were mixed with 1500 μL 1% formic acid/acetonitrile containing internal standard solution (solution ID H3304-1002, Human Metabolome Technologies, Inc., Tsuruoka, Japan) at 0 °C in order to inactivate enzymes. Then, the mixture was centrifuged at × 2300*g* for 5 min at 4 °C. The supernatant was filtered through Hybrid SPE phospholipid cartridge (55261-U; Supelco, Bellefonte, PA, USA) to remove phospholipids. The filtrate 400 μL was lyophilized and dissolved in 100 μL of 50% isopropanol/Milli-Q water solution for analysis. LC-TOFMS was performed using an Agilent LC System (Agilent 1200 series RRLC system SL) equipped with an Agilent 6230 time-of-light mass spectrometer (Agilent Technologies, Waldbronn, Germany). The systems were controlled by Agilent G2201AA ChemStation software version B.03.01 for CE (Agilent Technologies, Waldbronn, Germany). The cationic and anionic compounds were measured by using ODS column (2 × 50 mm, 2 μm).

Peaks were extracted using the MasterHands automatic integration software ver.2.16.0.15 (Keio University, Tsuruoka, Japan) to obtain peak information including the *m*/z ratio, migration time for (MT) for CE-TOF/MS measurement, retention time (RT) for LC-TOF/MS measurement and peak area. Signal peaks corresponding to “isotopomers,” “adduct ions,” and other “ions” of known metabolites were excluded. The remaining peaks were annotated with putative metabolites from the metabolite database and Known-Unknown library database established in the HMT, based on MT/RT and *m*/z ratio values as determined by TOF/MS. The tolerance range for the peak annotation was configured at ± 0.5 min for MT/RT, and ± 10 ppm for *m*/z ratio. In addition, the peak areas were normalized against those of the internal standards. Finally, the resultant relative area values were further normalized by sample amount. For metabolomics analysis, the relative area was defined as the relative concentration of each metabolite. The Human Metabolome Technologies, Inc., performed hierarchical cluster analysis (HCA) and principal component analysis (PCA) by our proprietary software, PeakStat and SampleStat, respectively. Detected metabolites were plotted on metabolic pathway maps using VANTED (Visualization and Analysis of Networks containing Experimental Data) software.

#### Health-promoting lifestyle profile (HPLP)

The HPLP was originally developed by Walker, Sechrist, and Pender in 1987 and revised as HPLP-II in 1995 [23]. Wei et al. developed the Japanese version of the HPLP-II and established its validity and credibility with a Cronbach’s *α* internal consistency coefficient of 0.90 [24]. The HPLP-II is a 52-item questionnaire composed of two main categories and six sub-dimension scales. The health-promoting behaviors category includes the health responsibility (9 items), physical activity (8 items), and nutrition (9 items) subscales. The psychosocial well-being category includes the spiritual growth (9 items), interpersonal relationship (9 items), and stress management (8 items) subscales. Each subscale can be used independently. Higher scores indicate a healthier lifestyle. All items on the HPLP-II are affirmative with no reverse questions. The answers were provided on a four-point Likert-type scale, and ratings of “never,” “sometimes,” “frequently,” and “regularly” were scored as 1, 2, 3, and 4 points, respectively. Therefore, the total scores on the HPLP-II ranged between 52 and 208. The scores on the “physical activity” and “stress management” subscales range between 8 and 32, and the scores on the other four subscales range between 9 and 36. In this study, we subsequently divided the total scores and the sum of each subscale score of the HPLP-II by the number of items; therefore, the mean item score ranged from 1 to 4. The internal consistency of the HPLP-II scale used in this study was demonstrated with a Cronbach’s *α* of 0.902. We divided the HPLP-II ratings into three groups of “good,” “moderate,” and “poor.” For the total HPLP-II scale, a score of 3.01–4 was considered “good,” a score of 2.01–3 was considered “moderate,” and a score of 1–2 was considered “poor.” All measurements obtained using the HPLP-II are summarized in Additional file 2.

#### Brief-type self-administered diet history questionnaire (BDHQ)

The BDHQ is a 58-item short version of the self-administered diet history questionnaire (DHQ) that assesses Japanese dietary habits. The BDHQ was reported to have a satisfactory ability to rank the energy-adjusted intakes of many nutrients in healthy Japanese adults [25]. The BDHQ assesses the dietary habits during the preceding month and consists of the following five sections: (i) intake frequency of 46 food and non-alcoholic beverage items; (ii) daily intake of rice, including the type of rice (refined or unrefined, etc.) and miso soup; (iii) frequency of drinking alcoholic beverages and the amount per drink of five different types of alcoholic beverages; (iv) usual cooking methods; and (v) general dietary behaviors. Most food and beverage items listed on the DHQ are very commonly consumed in Japan, with some modifications using a food list provided by the National Health and Nutrition Survey of Japan as additional information. Standard portion sizes and adult sizes of bowls for rice and cups for miso soup were derived from several recipe books for Japanese dishes. All measurements obtained using the BDHQ are summarized in Additional file 3.

### Empathy process scale

Empathy was evaluated using the Empathetic Process Scale, which was recently developed by Hayama et al. based on the Interpersonal Reactivity Index proposed by Davis [26]. This Japanese 30-item questionnaire was designed with six sub-dimension scores to assess both the cognitive and emotional aspects of empathy [27]. This scale focuses on more detailed emotional aspects than that developed by Davis. The emotional aspects of empathy include “Sharing positive emotions with others,” “Good feeling for others’ positive emotions,” “Sharing negative emotions with others,” and “Sympathy for others’ negative emotions.” The cognitive aspects of empathy include “Perspective taking” and “Sensibility about others’ emotions.” The self-reported responses were provide on a 5-point Likert-type scale with the anchors of 1 = Strongly disagree and 5 = Strongly agree. All six subscales consisted of five items with scores ranging from 5 to 25. The internal consistency of the Empathy Scale used in this study was demonstrated with a Cronbach’s *α* of 0.821. Two professional native-speaking English translators at Tokyo Kasei University translated the Japanese questionnaire into English (Additional file 5).

### Statistical analysis

All data are presented as medians with interquartile ranges (25–75th percentile). All statistical analyses were performed using SPSS version 19 (IBM Inc., Armonk, NY, USA) and GraphPad Prism 6.0 (GraphPad Software Inc., San Diego, CA, USA). Because the sample sizes in this study were relatively small (*n* = 10 in each group), we could not determine whether the transcripts, metabolites, blood, and psychological indices followed a normal distribution. Therefore, we chose the non-parametric Mann–Whitney *U* test to compare the detected values between the priest group and the control group. A *P* value of 0.05 or less was considered significant. The effect sizes (*r*) for the Mann–Whitney *U* test were calculated based on the *Z* value as described below. *N* represents the total sample size. Cohen’s guidelines indicate that a large effect size is *r* = 0.5, a medium effect size is *r* = 0.3, and a small effect size is *r* = 0.1 [28].

$$ r=\frac{Z}{\sqrt{N}} $$

To explore the significance of the deviated transcripts and metabolites in the “spiritual/religious” trained priests, Spearman’s rank correlation coefficients were calculated to assess the correlation between the psychological indices (Empathy Scales) and the significant transcriptional or metabolic candidates. Statistical significance was defined as *P* < 0.05.

## Results

### Descriptive characteristics of the participants’ lifestyle behaviors and blood chemistries

Lifestyle behaviors are considered essential components that influence well-being and health. Dietary habits, regular physical exercise, and religiosity/spirituality are major factors associated with these behaviors [29]. Therefore, we firstly compared the healthy lifestyle behaviors and dietary habits between the priests (*n* = 10) and the age-matched non-priest controls (*n* = 10). The healthy lifestyle behaviors and dietary habits were evaluated using the HPLP-II questionnaires and BDHQ questionnaires, respectively. The outcomes of the six HPLP-II sub-dimensions are dot-plotted in Additional file 4 and summarized in Additional file 2. Measurements from the BDHQ were summarized in Additional file 3. Additional files 2, 3 and 4 show that no significant differences were observed in the healthy lifestyle behaviors and daily nutrient intakes between the priests and the non-priest controls in this study.

Additional file 1 presents the serum chemistries of the participants in this study. The priests and the non-priest controls were matched with respect to age, gender (all males), ethnic group (all Japanese), and BMI, and there were no significant differences between the groups regarding these parameters (*P* > 0.05). In most of the blood indices, no significantly differences were observed at the basal state between the priests (*n* = 10) and the non-priest controls (*n* = 10). Only the sodium (Na) and potassium (K) concentrations were significantly different between the groups (*P* = 0.03 and 0.04, respectively); however, these values were within the normal healthy ranges. Two priests with diabetes had high blood sugar (133 and 164 mg/dL) and HbA1c (6.2 and 7.6%) levels; one priest also showed a high level of plasma creatinine (1.87 mg/dL) due to diabetic nephropathy. Notably, no significant difference (*P* = 0.47) in their levels of the systemic inflammatory marker C-reactive protein (CRP) was observed comparing to other healthy priests or all non-priest controls. The median CRP level in the priest group was 0.05 (0.03–0.08, 25–75%) (mg/dL) and that in the non-priest control group was 0.04 (0.03–0.05, 25–75%) (mg/dL). Notably, the results of the comparison of the serum nutritional variables between the groups shown in Additional file 1 (protein, cholesterol, triglyceride, glucose, Na, and K) were consistent with the findings obtained using the BDHQ (protein, cholesterol, fat, carbohydrate, sodium, and potassium) shown in Additional file 3.

### Microarray results and selection of candidate genes

To determine the gene expression profiles of the qualified priests at the basal state, we performed one-color-microarray experiments using Agilent platforms. We tested 50,599 probe sets to investigate the expression of 23,284 unique human genes.

To obtain an initial list of candidate genes, we performed a Ranked Product analysis to compare the ranks of the top differentially expressed genes between the priests and the controls. We set the threshold at a *P* value < 0.05; 162 probe sets representing 111 genes (42 up-regulated genes and 69 down-regulated genes) were differentially expressed between the priests and the non-priest controls after the probe sets passed filtering criteria of 5% false discovery rate (FDR). Additional file 6 provides a list of all 111 differentially expressed candidate genes. The 42 up-regulated genes and 69 down-regulated genes were subjected to a Gene Ontology (GO) analysis using AmiGO (http://amigo2.geneontology.org/amigo). The most significantly enriched functions include “immune effector process (GO:0002252),” “type I interferon signaling pathway (GO:0060337),” “cellular response to type I interferon (GO:0071357),” “innate immune response (GO:0045087),” “response to type I interferon (GO:0034340),” “defense response (GO:0006952),” and “defense response to virus (GO:0051607).”

Tables 1 and 2 show the expression values of representative ranked genes that were differentially expressed by more than 1.5-fold with their corresponding *P* values, FDRs, and average raw signals (*n* = 10, priests) in the microarray experiments. Table 1 lists the genes that were up-regulated in the priests, including type I IFN innate anti-viral response genes (e.g., MX1, RSAD2, IFIT1, IFIT3, IFI27, IFI44L, and E3 ubiquitin ligase (HERC5)), defensin4 (DEFA4), and soluble-type folate receptor (FOLR3). Table 2 lists the genes that were down-regulated in the priests, including hemoglobin γA (HBG1), keratin-associated protein (KRTAP10-12), sialic acid Ig-like lectin 14 (SIGLEC14), calcium-binding protein (S100P), and C17orf97.

**Table 1:** Up-expressed genes in the priests

Up genes	Enterz Gene ID	Gene name	Fold change	*P* value	FDR	Average raw
MX1	4599	MX dyn amin-like GTPase 1	2.00	5.7E − 05	0.0352	4503
IFI27	3429	Interferon-α-inducible protein 27	2.60	9.9E − 08	0.0003	1990
MYOM2	9172	Myomesin 2	1.84	6.6E − 08	0.0003	1957
IFIT3	3437	Interferon-induced protein with tetratricopeptide repeats 3	1.77	7.7E − 05	0.0410	1890
IFIT1	3434	Interferon-induced protein with tetratricopeptide repeats 1	2.20	5.8E − 06	0.0073	1682
RSAD2	91,543	Radical S-adenosyl methionine domain containing 2	2.99	< E − 09	0.0000	1347
FOLR3	2352	Folate receptor 3 (γ)	1.82	1.3E − 05	0.0129	1079
BTNL3	10,917	Butyrophilin-like 3	2.23	< E − 09	0.0000	880
DEFA4	1669	Defensin, α4, Corticostatin	2.09	2.1E − 06	0.0034	824
IFI44L	10,964	Interferon-induced protein 44-like	2.58	2.0E − 07	0.0005	726
BATF2	116,071	Basic leucine zipper transcription factor, ATF-like 2	1.54	8.7E − 05	0.0448	540
C4BPA	722	Complement component 4 binding protein, α	2.14	6.6E − 08	0.0003	353
CTSG	1511	Cathepsin G	1.73	7.2E − 05	0.0395	301
HERC5	51,191	HECT and RLD domain containing E3 ubiquitin protein ligase 5	2.05	2.0E − 05	0.0174	266
S100B	6285	S100 calcium binding protein B	1.52	4.5E − 05	0.0305	195
ARPC5	10,092	Actin related protein 2/3 complex, subunit 5, 16 kDa	1.66	2.4E − 05	0.0193	120
LOC 728715	728,715	Ovostatin homolog 2-like	2.75	3.3E − 07	0.0008	72
LOC 100509445	100,509,445	Uncharacterized LOC100509445	4.29	< E − 09	0.0000	67

**Fig. 1:**
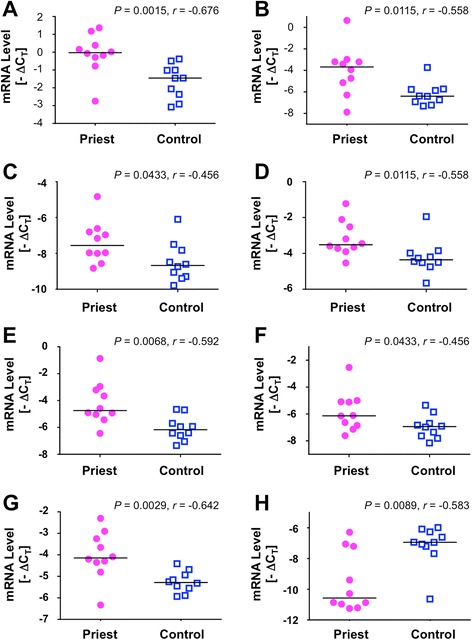
The dot plots of the representative transcriptional markers identified in the priests. Dots represent subjects (circle, priests; square, controls), and line represents median. Eight genes were selected for validation by quantitative real-time PCR (qRT-PCR). qRT-PCR data are normalized to the housekeeping gene GAPDH. Differences of each transcript were compared using a Mann–Whitney *U* test, indicating *P* value and the effect size *r*. **a** MX1 (*P* = 0.0015, *r* = − 0.676), **b** IFI27 (*P* = 0.0115, *r* = − 0.558), **c** IFIT1 (*P* = 0.0433, *r* = − 0.456), **d** IFIT3 (*P* = 0.0115, *r* = − 0.558), **e** RSAD2 (*P* = 0.0068, *r* = − 0.592), **f** IFI44L (*P* = 0.0433, *r* = − 0.456), **g** HERC5 (*P* = 0.0029, *r* = − 0.642), and **h** C17orf97 (*P* = 0.0089, *r* = − 0.583). Statistical significance was defined as *P* < 0.05. Cohen’s guidelines for the effect sizes (*r*) for Mann–Whitney *U* test are that a large effect is 0.5, a medium effect is 0.3, and small effect is 0.1 [28]

Representative list of genes up-expressed (> 1.5-fold) in priests compared to the controls. The described data is in descending ordered of the average raw signals (> 50) in the priests’ group microarray data. MX1, IFI27, IFIT1, IFIT3, RSAD2, IFI44L, and HERC5 have been validated by quantitative real-time PCR (Fig. 1)

**Table 2:** Down-expressed genes in the priests

Down genes	Enterz Gene ID	Gene name	Fold change	*P* value	FDR	Average raw
HBG1	3047	Hemoglobin, γA	0.40	6.6E − 08	0.0003	93,019
KRTAP 10-12	386,685	Keratin associated protein 10–12	0.65	1.7E − 05	0.0146	49,880
NPPA	4878	Natriuretic peptide A	0.64	4.0E − 05	0.0254	17,657
BPIFB2	80,341	BPI fold containing family B, member 2	0.60	2.6E − 05	0.0196	14,002
TUBB2A	7280	Tubulin, β2A class IIa	0.58	2.7E − 06	0.0055	8610
S100P	6286	S100 Ca^2+^ binding protein P	0.43	6.6E − 08	0.0003	5677
G3BP1	10,146	GTPase activating protein (SH3 domain) binding protein 1	0.66	2.9E − 05	0.0207	4888
NCR3	259,197	Natural cytotoxicity triggering receptor 3	0.58	9.8E − 05	0.0375	1941
SIGLEC14	100,049,587	Sialic acid Ig-like lectin 14	0.05	< E − 09	0.0000	1625
LAIR2	3904	Leukocyte-associated immunoglobulin-like receptor 2	0.62	7.1E − 05	0.0312	510
IGF2	3481	Insulin-like growth factor 2	0.57	7.4E − 05	0.0314	378
LOC 102467076	102,467,076	Uncharacterized LOC102467076	0.49	6.6E − 08	0.0004	347
RNF182	221,687	Ring finger protein 182	0.40	1.3E − 07	0.0000	140
C17orf97	400,566	Ligand of arginyltransferase 1 (LIAT1)	0.26	< E − 09	0.0000	121
C8orf17	100,507,249	Chromosome 8 open reading frame 17	0.40	1.6E − 05	0.0148	91
TEKT2	27,285	Tektin 2 (testicular)	0.59	5.3E − 05	0.0284	66
KCNJ18	100,134,444	Potassium inwardly rectifying channel subfamily J, member 18	0.65	3.6E − 05	0.0237	61
MDGA1	266,727	MAM domain containing glycosylphosphatidylinositol anchor 1	0.47	2.3E − 07	0.0007	54

Representative list of genes up-expressed (< 0.66-fold) in priests comparing to the controls. The described data is in descending ordered of the average raw signals (> 50) in the priests’ group microarray data. C17orf97 has been validated by quantitative real-time PCR (Fig. 1)

### Result validation by qRT-PCR

To further verify the microarray results using an independent technique, we performed quantitative real-time RT-PCR (qRT-PCR). We compared the basal expression levels of 12 selected representative transcripts in the priests, including MX1, RSAD2, IFIT1, IFIT3, IFI27, IFI44L, HERC5, DEFA4, FOLR3, HBG1, C17orf97, and S100P, between the priests and the non-priest controls. As shown in Fig. 1, the participant priests showed a significantly up-regulated expression of seven genes that are involved in type I IFN anti-viral responses (e.g., MX1, IFI27, IFIT1, IFIT3, RSAD2, IFI44L, and HERC5). C17orf97 was validated by qRT-PCR as a significantly down-regulated gene in the priests. This gene product is currently known as LIAT1 or ligand of arginyltransferase 1 (ATE1), because C17orf97 interacts with ATE1, which is a component of the N-end rule pathway of proteins degradation [30]. Notably, C17orf97 is also down-regulated in the hedonic well-being status [13]. No significant differences were observed in the expression levels of the remaining examined genes between the groups (*P* > 0.05).

### Metabolomics analysis

**Fig. 2:**
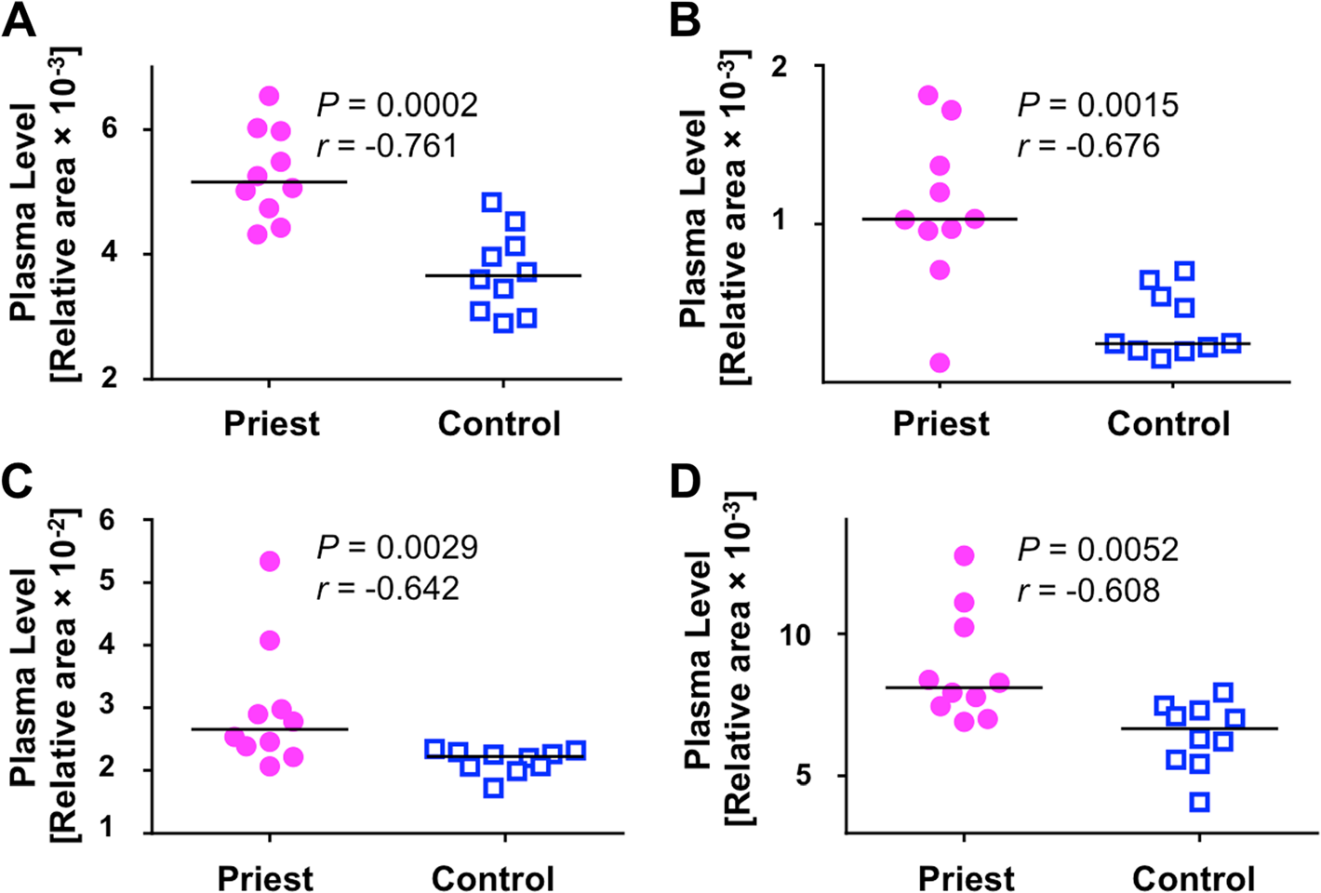
Representative four plasma metabolite markers identified in the priests. Dots represent subjects (circle, priests; square, controls), and line represents median. **a** Methionine in plasma of priests vs. the controls (*P* = 0.0002, *r* = − 0.761). **b** 3-Aminoisobutyric acid (BAIBA) in plasma of priests vs. the controls (*P* = 0.0015, *r* = − 0.676). **c** Phenylalanine in plasma of priests vs. the controls (*P* = 0.0029, *r* = − 0.642). **d** Choline in plasma of priests vs. the controls (*P* = 0.0052, *r* = − 0.608). Plasma level of each metabolite was shown as the relative area value calculated for metabolomics analysis by the Human Metabolome Technologies, Inc. The relative area value was defined as the relative concentration of each metabolite. Differences of each plasma metabolites were compared using a Mann–Whitney *U* test, indicating *P* value and the effect size *r*. Statistical significance was defined as *P* < 0.05. Cohen’s guidelines for the effect sizes (*r*) for Mann–Whitney *U* test are that a large effect is 0.5, a medium effect is 0.3, and small effect is 0.1 [28]

To clarify the underlying metabolic alterations in the spiritually trained Buddhists, we performed global metabolomics profiling to compare the priest participants with the non-priest controls. The complete data sets are shown in Additional file 7. In total, 275 metabolites were detected in the plasma of the participants as follows: 149 hydrophilic metabolites (105 and 44 metabolites in cationic and anionic modes, respectively) and 126 hydrophobic metabolites (62 and 64 metabolites in positive and negative modes, respectively). We detected 20 candidate metabolites in the priests, and the relative concentrations of these metabolites were significantly higher in the priests than those in the non-priest controls based on non-parametric Mann–Whitney *U* test (*P* < 0.05, Additional file 7). We chose 14 certain metabolites from the 20 candidate metabolites because these metabolite peaks were detected in all 20 participants. Table 3 showed the chosen metabolites in the priests, including 3-aminoisobutyric acid (BAIBA), choline, amino acids (methionine, phenylalanine, histidine, valine, isoleucine, and leucine), amino acid derivatives (symmetric dimethylarginine (SDMA), asymmetric dimethylarginine (ADMA), 2-aminoadipic acid (AABA)), creatine, and acylcarnitine (13:1). Figure 2 shows the following four representative metabolites that were significantly higher (*P* < 0.01) in the priests than in the non-priest controls: methionine (1.43-fold, *P* = 0.0002, *r* = − 0.761); BAIBA (3.06-fold, *P* = 0.0015, *r* = − 0.676); phenylalanine (1.36-fold, *P* = 0.0029, *r* = − 0.642); and choline (1.38-fold, *P* = 0.0052, *r* = − 0.608).

**Table 3:** Fourteen metabolite profiling in the priests

Metabolites	Priests (*n* = 10)	Control (*n* = 10)	Fold change	*P* value	*r*
Methionine	5.2E − 03 (4.7–6.0) E − 03	3.7E − 03 (3.1–4.1) E − 03	1.43	*0.0002*	− 0.761
3-Aminoisobutyric acid (BAIBA)	10.0E − 04 (9.6–13.7) E − 04	2.5E − 04 (2.0–5.4) E − 04	3.06	*0.0015*	− 0.676
Phenylalanine	2.7E − 02 (2.4–3.0) E − 02	2.2E − 02 (2.1–2.3) E − 02	1.36	0.0029	− 0.642
Choline	8.1E − 03 (7.5–10.2) E − 03	6.7E − 03 (5.6–7.3) E − 03	1.38	0.0052	− 0.608
SDMA (symmetric dimethylarginine)	2.2E − 04 (2.0–2.3) E − 04	2.0E − 04 (1.6–2.0) E − 04	1.20	0.010	− 0.563
2-Aminoadipic acid	3.3E − 04 (2.9–3.8) E − 04	2.0E − 04 (1.7–2.4) E − 03	1.45	0.012	− 0.558
2-Aminobutyric acid (AABA)	8.7E − 03 (7.6–10.7) E − 03	6.8E − 03 (5.9–7.3) E − 03	1.38	0.019	− 0.524
ADMA (asymmetric dimethylarginine)	1.8E − 04 (1.6–2.2) E − 04	1.5E − 04 (1.4–1.7) E − 04	1.20	0.025	− 0.507
Valine	10.3E − 02 (9.4–10.9) E − 02	9.0E − 02 (8.6–10.5) E − 02	1.09	0.023	− 0.507
AC(13:1)	5.9E − 05 (4.5–10.3) E − 05	3.2E − 05 (2.2–4.3) E − 05	1.79	0.027	− 0.491
Creatinine	1.3E − 02 (1.0–2.0) E − 02	0.9E − 02 (0.8–1.1) E − 02	1.64	0.030	− 0.483
Leucine	7.8E − 02 (7.5–9.2) E − 02	7.1E − 02 (6.8–7.8) E − 02	1.14	0.036	− 0.473
Histidine	2.3E − 02 (2.1–2.4) E − 02	2.0E − 02 (1.7–2.3) E − 02	1.10	0.043	− 0.456
Isoleucine	4.5E − 02 (4.2–5.2) E − 02	3.8E − 02 (3.4–4.4) E − 02	1.21	0.043	− 0.456

Values are expressed as median and interquartile range (25–75th percentile). A *P* value < 0.05 is statistically significant by Mann–Whitney *U* test. A *P* value < 0.05 is statistically significant by Mann–Whitney *U* test. Cohen’s guidelines for the effect sizes (*r*) for Mann–Whitney *U* test are that a large effect is 0.5, a medium effect is 0.3, and small effect is 0.1 [28]

### Distribution of empathy sub-dimension scores

We evaluated the psychological trait of empathy in the priests and the non-priest controls. Table 4 presents the empathy sub-dimension scores in the priests and the non-priest controls. The priest group exhibited significantly higher empathy in four sub-dimensions than the non-priest controls as follows: “Sharing positive emotions with others” (priests 22.0 vs. controls 18.0, *P* = 0.018, *r* = − 0.521); “Good feeling for others’ positive emotions” (priests 22.0 vs. controls 20.0, *P* = 0.029, *r* = − 0.487); “Sharing negative emotions with others” (priests 18.5 vs. controls 16.0, *P* = 0.039, *r* = − 0.462); and “Sensibility about others’ emotions” (priests 22.0 vs. controls 18.5, *P* = 0.042, *r* = − 0.455).

**Table 4:** Empathy scores in priests and the controls

Psychology	Priests (*n* = 10)	Control (*n* = 10)	*P* value	*r*
Sharing positive emotions with others	22.0 (18.0–24.0)	18.0 (15.0–20.0)	0.018	− 0.521
Good feeling for others’ positive emotions	22.0 (19.8–25.0)	20.0 (17.8–21.0)	0.029	− 0.487
Sharing negative emotions with others	18.5 (17.8–20.3)	16.0 (13.8–19.0)	0.039	− 0.462
Sympathy for others’ negative emotions	20.5 (19.5–23.5)	20.0 (18.8–20.0)	0.118	− 0.368
Sensibility about others’ emotions	22.0 (20.0–25.0)	18.5 (17.0–23.0)	0.042	0.455
Perspective taking	20.5 (18.5–25.0)	19.0 (17.3–20.0)	0.106	0.368

Values are expressed as median and interquartile range (25–75th percentile). A *P* value < 0.05 is statistically significant by Mann–Whitney *U* test. Cohen’s guidelines for the effect sizes (*r*) for Mann–Whitney *U* test are that a large effect is 0.5, a medium effect is 0.3, and small effect is 0.1 [28]

### Bivariate correlations (Spearman’s *ρ*) between the identified transcripts, metabolites, and the empathy sub-dimension scores

To examine the correlations among the identified transcripts, metabolites, and the four empathy sub-dimensions, we performed a correlation analysis using Spearman’s rank correlation coefficients (two-tailed) in the combined samples (*n* = 10 + 10: priests + non-priest controls). We observed a significant correlation (*ρ* = − 0.636 or 0.448 ~ 0.700) between the identified transcripts and metabolites (Table 5), between the identified transcripts and the four empathy sub-dimension scores (Table 6), and between the identified metabolites and the four empathy sub-dimension scores (Table 7).

**Table 5:** Bivariate correlations (Spearman’s *ρ*) between the transcriptional markers and metabolite markers

Transcriptional marker vs. metabolite marker	*ρ*	*P* value
C17orf97 vs. 3-amino isobutyric acid (BAIBA)	− 0.636	0.026
RSAD2 vs. 3-amino isobutyric acid (BAIBA)	0.571	0.008
HERC5 vs. 3-amino isobutyric acid (BAIBA)	0.559	0.010
MX1 vs. 3-amino isobutyric acid (BAIBA)	0.556	0.011
IFIT3 vs. 3-amino isobutyric acid (BAIBA)	0.516	0.020
IFIT1 vs. 3-amino isobutyric acid (BAIBA)	0.501	0.025
MX1 vs. methionine	0.538	0.014
IFI27 vs. methionine	0.492	0.028
MX1 vs. phenylalanine	0.480	0.032

**Table 6:** Bivariate correlations (Spearman’s *ρ*) between the transcriptional markers and empathy sub-dimension scores

Transcriptional marker vs. empathy	*ρ*	*P* value
HERC5 vs. sharing positive emotions with others	0.595	0.006
RSAD2 vs. sharing positive emotions with others	0.537	0.015
MX1 vs. sharing positive emotions with others	0.511	0.019
IFIT3 vs. sharing positive emotions with others	0.483	0.031
IFI44L vs. sharing positive emotions with others	0.479	0.033
C17orf97 vs. sharing positive emotions with others	− 0.459	0.042
C17orf97 vs. good feeling for other’s positive emotions	− 0.528	0.017
IFIT3 vs. sharing negative emotions with others	0.603	0.005
IFIT1 vs. sharing negative emotions with others	0.569	0.009
IFI44L vs. sharing negative emotions with others	0.536	0.015
HERC5 vs. sharing negative emotions with others	0.487	0.030
RSAD2 vs. sharing negative emotions with others	0.448	0.047
IFIT3 vs. sensibility about other’s emotions	0.496	0.026
HERC5 vs. sensibility about other’s emotions	0.470	0.037

**Table 7:** Bivariate correlations (Spearman’s *ρ*) between the metabolite markers and empathy sub-dimension scores

Metabolite marker vs. empathy	*ρ*	*P* value
3-Amino isobutyric acid (BAIBA) vs. sharing positive emotions with others	0.700	0.001
Asymmetric dimethylarginine (ADMA) vs. sharing positive emotions with others	0.602	0.005
Symmetric dimethylarginine (SDMA) vs. sharing positive emotions with others	0.561	0.010
Methionine vs. sharing positive emotions with others	0.477	0.033
Symmetric dimethylarginine (SDMA) vs. good feeling for other’s positive emotions	0.649	0.002
Asymmetric dimethylarginine (ADMA) vs. good feeling for other’s positive emotions	0.569	0.009
Histidine vs. good feeling for other’s positive emotions	0.491	0.028
3-Amino isobutyric acid (BAIBA) vs. good feeling for other’s positive emotions	0.455	0.044
Creatinine vs. sensitivity about other’s emotions	0.573	0.008

**Fig. 3:**
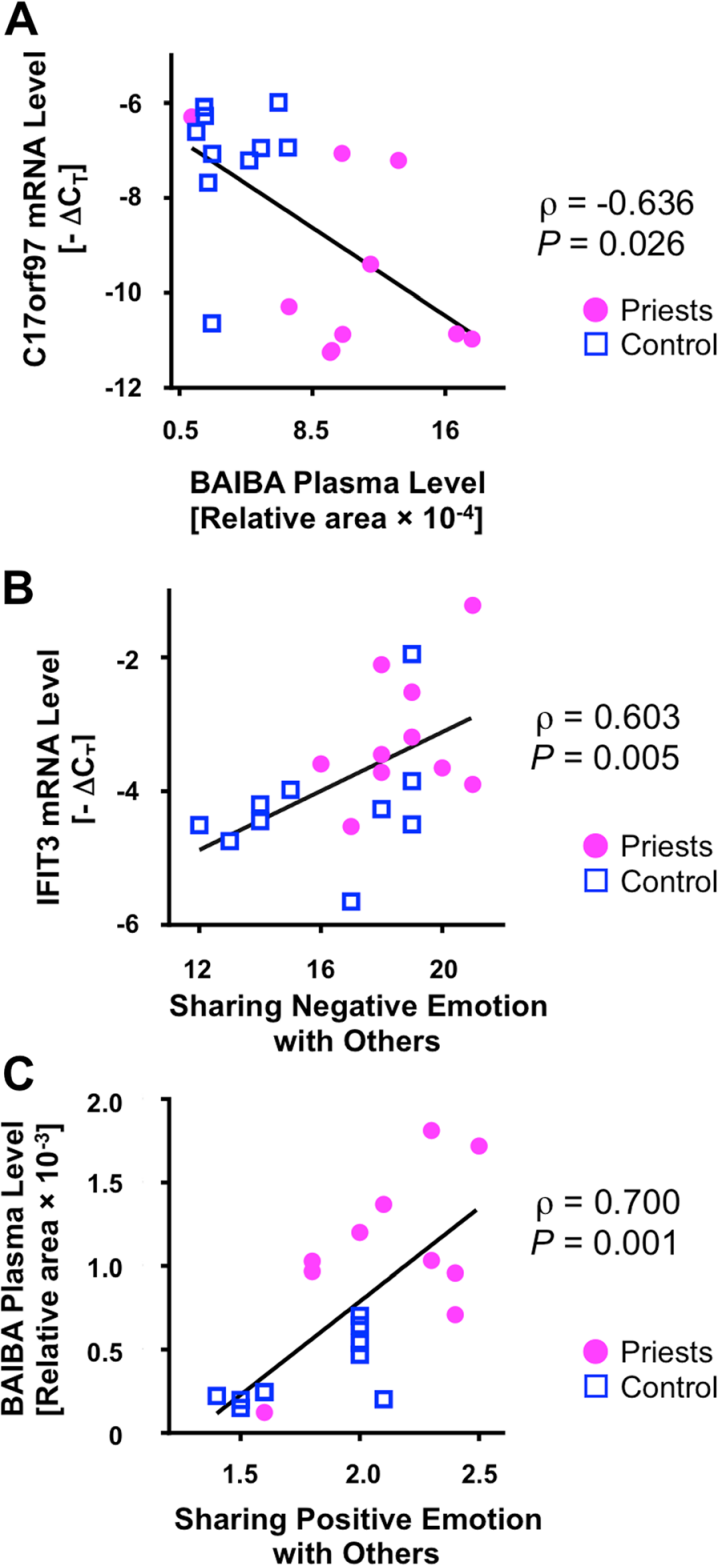
Scatter plots of representative correlation between the molecular markers identified in the priests and empathy. Spearman’s rank correlation coefficients were calculated. Dots represent subjects (circle, priests; square, controls). **a** C17orf97 transcript vs. 3-aminoisobutyric acid (BAIBA) metabolite (*ρ* = − 0.636). **b** IFIT3 transcript vs. empathy aspect “Sharing negative emotions with others” (*ρ* = 0.603). **c** 3-Aminoisobutyric acid (BAIBA) metabolite vs. empathy aspect “Sharing positive emotions with others” (*ρ* = 0.700)

As shown in Table 5, the BAIBA metabolite was significantly correlated with six distinct transcripts as follows: BAIBA was negatively correlated with C17orf97 (*ρ* = − 0.636, *P* = 0.026, Fig. 3) and positively correlated with five anti-viral genes including RSAD2 (*ρ* = 0.571, *P* = 0.008), HERC5 (*ρ* = 0.559, *P* = 0.010), MX1 (*ρ* = 0.556, *P* = 0.011), IFIT3 (*ρ* = 0.516, *P* = 0.020), and IFIT1 (*ρ* = 0.501, *P* = 0.025). Meanwhile, in addition to BAIBA, the MX1 gene was correlated with five different metabolites, including methionine, SDMA, phenylalanine, and glycodeoxycholic acid.

As shown in Table 6, “Sharing positive emotions with others” was associated with HERC5 (*ρ* = 0.595, *P* = 0.006), RSAD2 (*ρ* = 0.537, *P* = 0.015), MX1 (*ρ* = 0.511, *P* = 0.019), IFIT3 (*ρ* = 0.483, *P* = 0.031), IFI44L (*ρ* = 0.479, *P* = 0.033), and C17orf97 (*ρ* = − 0.459, *P* = 0.042), while “Sharing negative emotions with others” was associated with IFIT3 (*ρ* = 0.603, *P* = 0.005, Fig. 3), IFIT1 (*ρ* = 0.569, *P* = 0.009), IFI44L (*ρ* = 0.536, *P* = 0.015), HERC5 (*ρ* = 0.487, *P* = 0.030), and RSAD2 (*ρ* = 0.448, *P* = 0.047). “Sensitivity about others’ emotions” was correlated with IFIT3 (*ρ* = 0.496, *P* = 0.026) and HERC5 (*ρ* = 0.470, *P* = 0.037). “Good feeling for others’ positive emotions” was negatively correlated with C17orf97 (*ρ* = − 0.528, *P* = 0.017).

As shown in Table 7, “positive” emotion-related empathy was correlated with five distinct metabolites. “Sharing positive emotions with others” was associated with BAIBA (*ρ* = 0.700, *P* = 0.001, Fig. 3), ADMA (*ρ* = 0.602, *P* = 0.005), SDMA (*ρ* = 0.561, *P* = 0.010), and methionine (*ρ* = 0.477, *P* = 0.033). “Good feeling for others’ positive emotions” was associated with SDMA (*ρ* = 0.649, *P* = 0.002), ADMA (*ρ* = 0.569, *P* = 0.009), histidine (*ρ* = 0.491, *P* = 0.028), BAIBA (*ρ* = 0.455, *P* = 0.044). Finally, “Sensibility about others’ emotions” was positively related with creatinine (*ρ* = 0.573, *P* = 0.008).

## Discussion

Spirituality/religiosity is one of the unique aspects of human social environments and can be positively associated with psychological and physical health according to psycho-neuro-immune models of health regulation [1]. Our current cross-sectional study defined the systemic signatures of the gene expression and the metabolic profiles in Buddhist priests compared with those in the non-priest controls. The list of identified transcripts included components of type I IFN responses involved in innate anti-viral protection. Additionally, the identified metabolites indicated that enhanced proteolytic metabolism might occur in the priests. In contrast, no significant differences were observed in the healthy lifestyle behaviors and daily nutrient intake between the two groups. Interestingly, we observed a significant correlation between empathy and the molecular markers identified in the priests.

Among the top ranked list of significantly up-regulated transcripts in the priests, seven gene products (MX1, RSAD2, IFIT1, IFIT3, IFI27, IFI44L, and HERC5) were involved in anti-viral protection mechanisms with type I IFN signaling [31–33]. It is unclear whether the observed basal physiological state in the priest participants controls inflammatory responses or the pathological inflammatory states. However, acute infectious illnesses or chronic inflammation likely did not cause the up-regulation of the type I IFN responsive genes in the priests because no apparent infection signs and symptoms were observed in any of the participants on the day of the experiment, and no significant difference were detected in the plasma CRP levels (*P* = 0.47), which is a systemic inflammatory marker, between the priests and the non-priest controls (Additional file 1). Notably, the fold change value of these seven transcripts (1.77- to 2.99-fold) seemed to be within the normal range of physiological variations and were one-digit less than those that are observed under infection conditions [34, 35]. Furthermore, no significant differences were detected between the two groups in the expression of inflammation-related genes. One unique feature of the type I IFN system is a weak signal that constitutively produces IFN-α, which is critical to “rev-up” for efficient and robust responses to viral infections in innate immune cells [36]. The up-regulation of the seven genes might possibly provide a foundation for more efficient or sensitive cellular responses for anti-viral protection. “Trained immunity” was recently described as a concept related to the memory-like innate immune function after microbial encounters [37]. Repeated exposure to specific environments or conditions during common religious/spiritual practices might trigger long-lasting changes as “allostatic responses” [38] in the anti-viral response of the type I IFN pathways. Furthermore, the trained spiritual/religious priests may have conditioned themselves to respond more quickly or with a higher sensitivity to environmental changes, particularly viral infections. Notably, six of the seven identified anti-viral genes were inversely down-regulated under socially adverse conditions, such as chronic loneliness [9, 10]. In this pilot study, we did not exclude three priests with known physical ailments from statistical analyses due to the limited number of recruited priests. Nonetheless, the results of analyses performed when these priests were excluded exhibited similar significances (Additional file 8).

We uncovered that Buddhists had higher levels of plasma free amino acids and amino acid-derivatives than the non-priest controls. These results indicated that the Buddhists might exhibit higher intracellular protein turnover/metabolism. The two major systems involved in cellular proteolysis are the ubiquitin-proteasome system [39] and the autophagy system [40]. Both systems play crucial roles in adapting and properly sustaining protein turnover (i.e., proteostasis) under a large variety of different environmental conditions. In particular, the autophagy pathway plays a crucial role in the resistance against infections and inflammatory conditions [41]. Therefore, autophagy may mediate the cytoprotective processes by maintaining protein and organelle quality control in the system better in the Buddhists than in the non-priest-controls. Among the identified plasma metabolites, BAIBA was correlated with the expression of six transcripts, including C17orf97, RSAD2, HERC5, MX1, IFIT3, and ITIF1 (Table 5). Furthermore, BAIBA can be generated by the catabolism of the branched-chain amino acid valine and functions as a myokine secreted from the skeletal muscle cells to alter the functions of other tissues. For example, BAIBA increases the expression of brown-adipocyte-specific genes in white adipocytes and β-oxidation genes in hepatocytes through a peroxisome proliferator-activated receptor alpha (PPARα)-dependent mechanism [42]. Although we cannot determine a causal relationship responsible for the observed correlations, we speculate that circulating BAIBA may have greater effects on leukocyte gene expression. Future studies are needed to determine the direct action of BAIBA on the up-regulation of anti-viral genes in peripheral leukocytes.

Choline, which is another notable metabolite in the priests, serves as a component in blood and membrane phospholipids and structural lipoproteins and is a precursor for the neurotransmitter acetylcholine. Choline and its oxidative product betaine, which serves as a methyl group donor, are important sources of one-carbon units. In this study, the priests showed significantly higher levels of plasma choline (8.1E − 3 vs. 6.7E − 3: 1.38-fold, *P* = 0.0052, *r* = − 0.608, Table 3) than the non-priest controls, and their levels of betaine were higher (2.7E − 2 vs. 2.3E − 2: 1.17-fold, *P* = 0.052, Additional file 7). Choline promotes homocysteine remethylation to methionine and affects the concentration of the universal methyl donor S-adenosylmethionine (SAM). Altered concentrations of SAM may influence DNA methylation at cytosine bases, thereby influencing gene transcription, genomic imprinting, and genomic stability [43]. Plasma choline is delivered across the blood-brain-barrier by a specific transporter [44]. Therefore, in this study, the higher levels of plasma choline likely increased the brain choline levels in the priests compared with that in the non-priest controls. Experimental studies suggest that increased levels of brain choline and phosphatidylcholine improve memory performance and cognitive function as well as enhance neuroprotection and neurorepair activities [45]. Additionally, plasma choline levels are inversely associated with high anxiety levels [46]. Altogether, we hypothesized that the higher levels of plasma choline in the priests may exhibit some beneficial effects on the neuropsychological status, such as improvements in cognitive function and reduced anxiety. Troen et al. showed that cognitive dysfunction in folate-deficient rats was related to depletion of phosphatidylcholine in the brain. Notably, dietary methionine could prevent both cognitive impairment and low phosphatidylcholine [47]. Therefore, the higher levels of plasma methionine in the priests in this study could also present further beneficial contributions to cognitive functions.

The highlight of this study was that we observed a significant correlation between empathy and the molecular markers identified in the priests (Tables 6 and 7). The Buddhist priests showed higher levels of empathy than the non-priest control (Table 4). Previous studies have shown that religious people tend to perceive themselves as pro-social and report higher levels of altruism or charitable deeds compared with non-religious people [5]. Individual Buddhists lead their own daily lives according to Buddhism values and virtues. Compassion (or loving-kindness) is a fundamental Buddhist concept of inter-personal relationships and is defined as the deep wish to relieve others’ suffering, coupled with the motivation to alleviate such suffering [17]. Empathy is a core element of both compassion (the wish to relieve others’ suffering) and loving-kindness (the wish of happiness for others) and an affective response that arises from the comprehension of another’s emotional state [18]. Although compassion is interpersonal, compassion has also been empirically linked to personal benefits, including increased positive emotions [48], improved physical health [49], and a reduced immunological stress response [50]. Emerging literature suggests that positive emotional styles (higher activation/arousal of positive emotions) are associated with improved protective immune responses [51, 52]. Notably, this study also showed that the positive emotional aspect of empathy is significantly correlated with the five transcripts for anti-viral responses (IFIT3, HERC5, RSAD2, IFI44L, and MX1) as shown in Table 6. Meanwhile, C17orf97, which was a down-regulated gene in the priests, and three representative metabolites, i.e., BAIBA, SDMA, and ADMA, were significantly correlated with the positive empathy aspects of “Sharing the positive emotions with others” and “Good feeling for others’ positive emotions” (Tables 6 and 7). The current results are the first to demonstrate a link between the molecular signatures of Buddhist priests and the psychological aspects of empathy.

Finally, a large body of research indicates that lifestyle interventions such as physical exercise and a healthy diet are effective for inducing phenotypic alterations in circulating cells and influencing immune function as a non-clinical human model of the body’s response to physiological stresses [53]. Additionally, both physical exercise and a healthy diet influence the human gene expression profiles in leukocytes [54, 55]. In the current pilot study, no significant differences in dietary habits and physical activity were observed between the priests and the non-priest controls (Additional files 2, 3 and 4). Despite the small sample sizes (*n* = 10 in each group), the scores on the overall HPLP-II in both groups were within the ranges obtaining in 512 male residents (mean age 45.5 ± 13.1 years) in a mixed rural-urban representative area in Japan. The actual states of the community residents’ (*n* = 1176: 512 males and 664 females) lifestyles were recently investigated using the same HPLP II questionnaires used in this study [24].

### Limitations

The identified transcript and metabolite profiles in the priest participants may potentially serve as markers and mediators for daily spiritual/religious practices. However, this study had several limitations. First, the sizes of the participant groups were too small to generalize the observed results. Second, the “cross-sectional design” made it impossible to determine the cause-and-effect relationship among the identified markers (transcriptomics and metabolomics), and spiritual/religious practices, or the empathy aspects. Third, the psychological aspects of empathy were based on subjective self-reports, leading to a potential recall bias, which could have influenced the accuracy of the reported data. Fourth, no direct biological measures of immune cells were available in this study (e.g., NK cell or dendritic cell activities or effector responses to an immunologic challenge). Finally, the priests selected for this study were from only one religious affiliation, Shingon Buddhism; therefore, we must compare the basal transcriptome/metabolome profiles of other religious priests carefully to generalize these results. Despite the relatively small sample size, we were able to detect the inter-individual consistency of the transcriptional/metabolic alterations in the Buddhist priests. In the future, it will be important to determine whether Buddhists acquire the representative transcriptome and metabolome profiles at baseline or during the course of training needed to become a prospective Buddhist priest [19]. Fancourt et al. recently demonstrated that group-drumming interventions produce psychological benefits associated with a shift toward an anti-inflammatory immune profile [56]. Therefore, we need to examine the correlations between the identified molecular markers and other characteristic factors of daily Buddhist practices (e.g., the number of Buddhist prayers one offers each day or the number of hits of a Buddhist drum each day). The up-regulated set of anti-viral responsive genes may indicate that the trained priests have gained unique immunological traits to protect their bodies from environmental parasitic infections based on the “behavioral-immune response hypothesis.”

## Conclusions

In this pilot study, we integrated in vivo phenotyping with transcriptomics, metabolomics, and psychological analyses, thereby identifying distinguishing biological characteristics and their association with empathy in Buddhism priests. The identification of the distinct transcripts and metabolites in the priests may be a first step toward understanding the molecular context of systemic biological alterations in the unique signature of spirituality/religiosity in Buddhists.

## Additional files

Additional file 1: Physiological characteristics of all participants. Values are expressed as median and interquartile range (25–75th percentile). A *P* value < 0.05 is statistically significant by Mann–Whitney *U* test. Cohen’s guidelines for the effect sizes (*r*) for Mann-Whitney *U* test are that a large effect is 0.5, a medium effect is 0.3, and small effect is 0.1 (Fritz et al. 2012). BMI, body mass index; T-protein, total protein; AST, aspartate transaminase; ALT, alanine transaminase; γ-GT, gamma-glutamyl transferase; CRP, C-reactive protein; HDL-Chol, high-density lipoprotein-cholersterol; LDL-Chol, low-density lipoprotein-cholesterol; BUN, blood urea nitrogen; HbA1c, hemoglobin A1c; RBC, red blood cell count; WBC, white blood cell count; MCV, mean corpuscular volume; MCH, mean corpuscular hemoglobin; MCH-C, mean corpuscular hemoglobin concentration; PLT-C, platelet count. (DOCX 533 kb)

Additional file 2: Comparison of health-promoting behaviors between the priests and the controls estimated by health-promoting lifestyle profile-II (HPLP-II). Values are expressed as median and interquartile range (25–75th percentile). A *P* value < 0.05 is statistically significant by Mann–Whitney *U* test. Cohen’s guidelines for the effect sizes (*r*) for Mann–Whitney *U* test are that a large effect is 0.5, a medium effect is 0.3, and small effect is 0.1 [28]. (DOCX 27 kb)

Additional file 3: Comparison of mean daily energy intake and crude and energy-adjusted nutrient intakes estimated by BDHQ between the priests and the controls. Representative mean daily crude nutrient intakes were estimated by BDHQ. Values are expressed as median and interquartile range (25–75th percentile). A *P* value < 0.05 is statistically significant by Mann–Whitney *U* test. Cohen’s guidelines for the effect sizes (*r*) for Mann-Whitney *U* test are that a large effect is 0.5, a medium effect is 0.3, and small effect is 0.1 [28]. (DOCX 535 kb)

Additional file 4: Comparisons of health-promoting lifestyle profiles (HPLP-II) between the priests and the controls. HPLP-II is a 52-item questionnaire composed of two main categories and six sub-dimension scales. The health-promoting behaviors category includes health responsibility, physical activity, and nutrition subscales. The psychosocial well-being category includes spiritual growth, interpersonal relationship, and stress management subscales. Dots represent subjects (●, priests *n* = 10; □, controls *n* = 10), and line represents median. Differences of each sub-dimension of HPLP-II were compared using a Mann–Whitney *U* test, indicating *P* value and the effect size *r*. (A) health responsibility (*P* = 0.864, *r* = −0.042), (B) physical activity (*P* = 0.837, *r* = −0.051), (C) nutrition (*P* = 0.423, *r* = −0.187), (D) spiritual growth (*P* = 0.678, *r* = −0.103), (E) interpersonal relationship (*P* = 0.615, *r* = −0.119), (F) stress management (*P* = 0.593, *r* = −0.127). Statistical significance was defined as *P* < 0.05. Cohen’s guidelines for the effect sizes (*r*) for Mann–Whitney *U* test are that a large effect is 0.5, a medium effect is 0.3, and small effect is 0.1 [28]. (TIFF 260 kb)

Additional file 5: The empathetic process scale questionnaire. (DOCX 526 kb)

Additional file 6: The list of distinct transcripts identified in the priests. (XLSX 22 kb)

Additional file 7: The metabolome analysis. (XLSX 136 kb)

Additional file 8: The statistical analysis without data of three priest participants with known physical ailments (priest, *n* = 7 vs. non-priest controls; *n* = 10). Values are expressed as median and interquartile range (25–75th percentile). A *P* value < 0.05 is statistically significant by Mann–Whitney *U* test. A *P* value < 0.05 is statistically significant by Mann–Whitney *U* test. Cohen’s guidelines for the effect sizes (*r*) for Mann–Whitney *U* test are that a large effect is 0.5, a medium effect is 0.3, and small effect is 0.1 [28]. (XLSX 41 kb)

## Abbreviations

AABA: 2-Aminoadipic acid
ADMA: Asymmetric dimethyl-arginine
ATE1: Arginyltranseferase 1
BAIBA: 3-Aminoisobutylic acid
BDHQ: A brief-type self-administered diet history questionnaire
CRP: C-reactive protein
DEFA4: Defensin alpha 4
FOLR3: Folate receptor 3
HBG1: Hemoglobin γA
HERC5: HECT and RLD domain containing E3 ubiquitin protein ligase 5
HPLP-II: The Health-Promoting Lifestyle Profile-II
IFI44L: Interferon induced protein 44 like
IFIT: Interferon induced protein with tetratricopeptide repeats
IFN: Interferon
IL: Interleukin
KRTAP10-12: Keratin-associated protein
LIAT1: Ligand of arginyltransferase 1
MX1: MX dynamin-like GTPase 1
NK: Natural killer
PPARα: Peroxisome proliferator-activated receptor alpha
RSAD2: Radical S-adenosyl methionine domain containing 2
SIGLEC14: Sialic acid Ig-like lectin 14
SMDA: Symmetric dimethyl-arginine
TNF: Tumor necrosis factor
