# Supplementary material for: Distinct transcriptional and metabolic profiles associated with empathy in Buddhist priests: a pilot study
Source: Hum Genomics. 2017 Sep 2;11:21. doi: 10.1186/s40246-017-0117-3 (PMC5581455; doi:10.1186/s40246-017-0117-3)
Supplement: Supplementary file 1 — Physiological characteristics of all participants. Values are expressed as median and interquartile range (25–75th percentile). A P value < 0.05 is statistically significant by Mann–Whitney U test. Cohen’s guidelines for the effect sizes (r) for Mann-Whitney U test are that a large effect is 0.5, a medium effect is 0.3, and small effect is 0.1 (Fritz et al. 2012). BMI, body mass index; T-protein, total protein; AST, aspartate transaminase; ALT, alanine transaminase; γ-GT, gamma-glutamyl transferase; CRP, C-reactive protein; HDL-Chol, high-density lipoprotein-cholersterol; LDL-Chol, low-density lipoprotein-cholesterol; BUN, blood urea nitrogen; HbA1c, hemoglobin A1c; RBC, red blood cell count; WBC, white blood cell count; MCV, mean corpuscular volume; MCH, mean corpuscular hemoglobin; MCH-C, mean corpuscular hemoglobin concentration; PLT-C, platelet count. (DOCX 533 kb) [file 40246_2017_117_MOESM1_ESM.docx]

Table S1_Ohnishi_Empathy Associated transcripts and metabolites.

|  |  | **Priest** | **Control** | ***P* value** | ***r*** |
| --- | --- | --- | --- | --- | --- |
| Number |  | 10 | 10 |  |  |
| Gender | Male | 10 | 10 |  |  |
| Age | Years | 38.5 (30.0–47.0) | 33.5 (31.0–42.5) | 0.60 | -0.120 |
| BMI |  | 23.6 (22.8–26.2) | 20.9 (19.7–23.8) | 0.06 | -0.420 |
| T-Protein | g/dL | 7.4 (7.0–7.6) | 7.3 (6.9–7.5) | 0.60 | -0.120 |
| AST | U/L | 18 (16–23) | 18 (15–21) | 0.91 | -0.025 |
| ALT | U/L | 19 (15–22) | 20 (17–27) | 0.32 | -0.220 |
| γ-GT | U/L | 23 (16–39) | 20 (18–32) | 0.60 | -0.120 |
| CRP | mg/dL | 0.05 (0.03–0.08) | 0.04 (0.03–0.05) | 0.47 | -0.160 |
| Cholesterol | mg/dL | 201 (161–220) | 186 (162–214) | 0.73 | -0.076 |
| HDL-Chol. | mg/dL | 52 (48–62) | 57 (42–61) | 0.97 | -0.008 |
| LDL-Chol. | mg/dL | 127 (91–138) | 120 (90–137) | 0.73 | -0.076 |
| Triglyceride | mg/dL | 125 (81–252) | 103 (72–199) | 0.52 | -0.144 |
| Ureic acid | mg/dL | 5.7 (4.6–6.1) | 6.2 (4.9–7.0) | 0.24 | -0.263 |
| BUN | mg/dL | 14 (10–17) | 11 (10–13) | 0.16 | -0.316 |
| Creatinine | mg/dL | 0.7 (0.7–1.0) | 0.8 (0.7–0.8) | 0.52 | -0.144 |
| Na | mmol/L | 141 (140–142) | 143 (142–145) | 0.03 | -0.481 |
| K | mmol/L | 4.8 (4.5–5.1) | 4.5 (4.1–4.7) | 0.04 | -0.468 |
| Cl | mmol/L | 104 (103–108) | 106 (104–107) | 0.40 | -0.188 |
| Albumin | g/dL | 4.6 (4.5–4.8) | 4.6 (4.4–4.8) | 0.65 | -0.102 |
| Glucose | mg/dL | 95 (79–141) | 85 (82–91) | 0.34 | -0.102 |
| HbA1c | % | 5.4 (5.2–6.6) | 5.4 (5.2–5.4) | 0.45 | -0.170 |
| RBC | ×10^4^/µL | 512 (465–556) | 497 (479–517) | 0.50 | -0.152 |
| WBC | ×10^3^/µL | 5.3 (3.9–7.5) | 5.1 (4.6–7.7) | 0.73 | -0.072 |
| Hemoglobin | g/dL | 16.0 (14.6–16.9) | 15.7 (14.7–16.1) | 0.60 | -0.119 |
| Hematocrit | % | 45.7 (43.1–49.0) | 45.4 (42.6–47.4) | 0.45 | -0.169 |
| MCV | fL | 90 (88–91) | 89 (87–95) | 0.91 | --0.025 |
| MCH | pg | 31.0 (30.1–31.3) | 31.0 (30.2–32.2) | 0.50 | -0.152 |
| MCH-C | g/dL | 34.0 (33.6–34.9) | 34.0 (33.8–34.9) | 0.38 | -0.195 |
| PLT-C | ×10^4^/µL | 24.0 (22.1–28.9) | 25.0 (21.7–27.3) | 0.82 | -0.051 |
