# Supplementary material for: Distinct transcriptional and metabolic profiles associated with empathy in Buddhist priests: a pilot study
Source: Hum Genomics. 2017 Sep 2;11:21. doi: 10.1186/s40246-017-0117-3 (PMC5581455; doi:10.1186/s40246-017-0117-3)
Supplement: Supplementary file 2 — Comparison of health-promoting behaviors between the priests and the controls estimated by health-promoting lifestyle profile-II (HPLP-II). Values are expressed as median and interquartile range (25–75th percentile). A P value < 0.05 is statistically significant by Mann–Whitney U test. Cohen’s guidelines for the effect sizes (r) for Mann–Whitney U test are that a large effect is 0.5, a medium effect is 0.3, and small effect is 0.1 [28]. (DOCX 27 kb) [file 40246_2017_117_MOESM2_ESM.docx]

Table S2_Ohnishi_Empathy Associated transcripts and metabolites.

|  | Priests  (n = 10) | Control  (n = 10) | *P* value | *r* |
| --- | --- | --- | --- | --- |
| Health responsibility | 2.40 (1.70–3.03) | 2.25 (1.90–2.80) | 0.864 | -0.042 |
| Physical activity | 1.90 (1.48–2.43) | 2.20 (1.40–2.40) | 0.837 | -0.051 |
| Nutrition | 2.65 (2.23–2.83) | 2.40 (2.03–2.75) | 0.423 | -0.187 |
| Spiritual growth | 2.85 (2.63–3.10) | 2.80 (2.55–2.98) | 0.678 | -0.103 |
| Interpersonal relationship | 2.95 (2.38–3.25) | 2.80 (2.70–2.90) | 0.615 | -0.119 |
| Stress management | 2.70 (2.18–2.95) | 2.50 (2.33–2.85) | 0.593 | -0.127 |
| Total score of HPLP-II | 2.50 (2.28–2.88) | 2.45 (2.30–2.80) | 0.895 | -0.076 |
