# Supplementary material for: Distinct transcriptional and metabolic profiles associated with empathy in Buddhist priests: a pilot study
Source: Hum Genomics. 2017 Sep 2;11:21. doi: 10.1186/s40246-017-0117-3 (PMC5581455; doi:10.1186/s40246-017-0117-3)
Supplement: Supplementary file 3 — Comparison of mean daily energy intake and crude and energy-adjusted nutrient intakes estimated by BDHQ between the priests and the controls. Representative mean daily crude nutrient intakes were estimated by BDHQ. Values are expressed as median and interquartile range (25–75th percentile). A P value < 0.05 is statistically significant by Mann–Whitney U test. Cohen’s guidelines for the effect sizes (r) for Mann-Whitney U test are that a large effect is 0.5, a medium effect is 0.3, and small effect is 0.1 [28]. (DOCX 535 kb) [file 40246_2017_117_MOESM3_ESM.docx]

Table S3_Ohnishi_Empathy Associated transcripts and metabolites.

|  |  | **Priest** | **Control** | ***P* value** | **r** |
| --- | --- | --- | --- | --- | --- |
| Protein | g/day | 68.5 (43.7–88.1) | 77.3 (59.6–91.0) | 0.436 | -0.186 |
| Fat | g/day | 46.6 (37.2–71.4) | 66.0 (53.3–76.2) | 0.143 | -0.338 |
| Saturated fat | g/dL | 11.7 (8.6–20.6) | 17.5 (15.3–22.5) | 0.166 | -0.321 |
| Monounsaturated fat | g/day | 17.2 (12.9–24.9) | 22.9 (18.9–27.9) | 0.109 | -0.364 |
| Polyunsaturated fat | g/day | 12.2 (8.3–14.9) | 15.8 (12.5–17.8) | 0.166 | -0.321 |
| n-6 polyunsaturatede fat | g/day | 10.1 (6.9–11.8) | 13.0 (10.1–15.1) | 0.166 | -0.321 |
| n-3 polyunsaturated fat | g/day | 1.94 (1.40–3.09) | 2.60 (2.28–2.96) | 0.382 | -0.203 |
| Cholesterol | mg/day | 305 (190–435) | 361 (270–556) | 0.315 | -0.237 |
| Carbohydrate | g/day | 228 (172–295) | 252 (227–310) | 0.481 | -0.169 |
| Total dietary fiber | g/day | 10.49 (5.26–20.42) | 10.10 (7.58–15.42) | 0.971 | -0.017 |
| Soluble dietary fiber | g/day | 2.84 (1.27–5.36) | 2.53 (1.66–4.06) | 0.971 | -0.017 |
| Insoluble dietary fiber | g/day | 7.53 (3.87–14.02) | 7.27 (5.66–10.81) | 0.912 | -0.034 |
| Alcohol | g/day | 3.03 (0.0–29.69) | 0.72 (0.0–8.87) | 0.677 | -0.104 |
| Retinol | μg/day | 256 (121–645) | 400 (245–642) | 0.353 | -0.220 |
| Vitamin A  (retinol equivalent) | μg/day | 626 (282–1047) | 716 (398–1000) | 0.579 | -0.135 |
| α-carotene | μg/day | 138 (14–359) | 158 (74–362) | 0.481 | -0.169 |
| β-carotene equivalent | μg/day | 2600 (1294–5458) | 2889 (1869–4833) | 0.631 | -0.118 |
| Cryptoxanthin | μg/day | 294 (163–459) | 224 (74–318) | 0.218 | -0.287 |
| α-tocopherol | mg/day | 6.62 (3.73–9.06) | 7.63 (6.44–10.12) | 0.393 | -0.203 |
| Vitamin K | μg/day | 321 (134–654) | 290 (178–504) | 0.912 | -0.034 |
| Thiamin | mg/day | 0.76 (0.49–1.04) | 0.82 (0.65–1.00) | 0.671 | -0.102 |
| Riboflavin | mg/day | 1.52 (0.70–1.98) | 1.42 (0.96–1.89) | 0.897 | -0.034 |
| Niacin | mgNE/day | 15.2 (10.1–21.0) | 18.2 (14.1–21.9) | 0.481 | -0.169 |
| Vitamin B6 | mg/day | 1.20 (0.71–1.78) | 1.13 (0.93–1.36) | 0.853 | 0.051 |
| Vitamin B12 | μg/day | 6.15 (4.21–9.07) | 7.64 (4.81–9.93) | 0.684 | -0.102 |
| Folate | μg/day | 343 (148–506) | 300 (202–416) | 0.912 | -0.034 |
| Pantothenic acid | mg/day | 7.19 (4.11–8.91) | 6.71 (5.53–8.47) | 0.971 | -0.017 |
| Vitamin C | mg/day | 82 (43–154) | 91 (70–113) | 0.853 | -0.051 |
| Sodium | mg/day | 3741 (3190–5431) | 4900 (3633–5682) | 0.190 | -0.304 |
| Potassium | mg/day | 2371 (1494–3758) | 2387 (1818–3243) | 0.971 | -0.017 |
| Zinc | mg/day | 7.97 (5.07–10.31) | 9.10 (7.20–10.13) | 0.436 | -0.186 |
| Copper | mg/day | 1.00 (0.69–1.45) | 1.07 (0.88–1.32) | 0.739 | -0.085 |
| Manganes | mg/day | 2.43 (1.48–3.45) | 2.87 (2.24–3.74) | 0.48 | -0.169 |
