# Supplementary figures and images for: Distinct transcriptional and metabolic profiles associated with empathy in Buddhist priests: a pilot study
Source: Hum Genomics. 2017 Sep 2;11:21. doi: 10.1186/s40246-017-0117-3 (PMC5581455; doi:10.1186/s40246-017-0117-3)

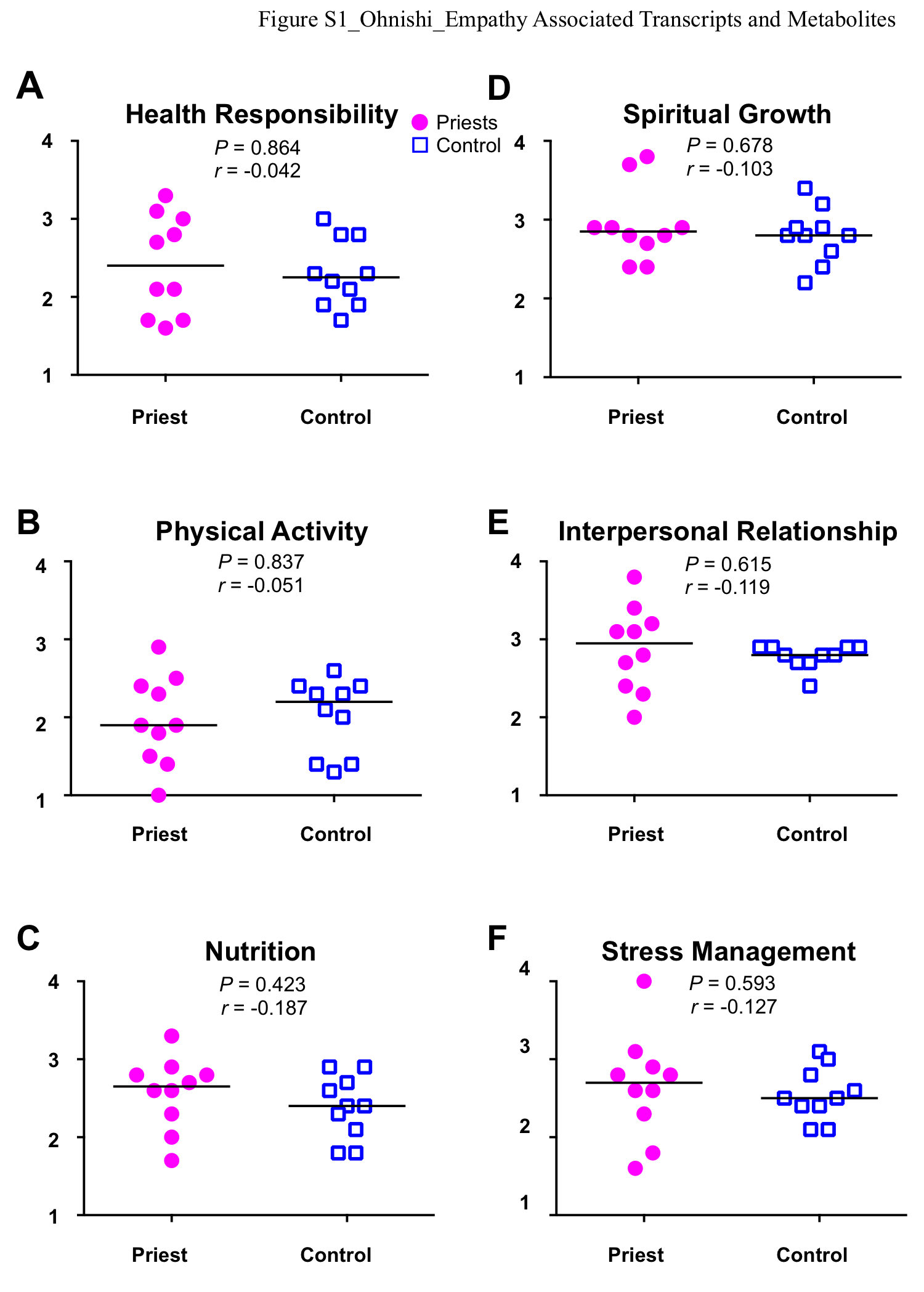

Supplement: Supplementary file 4 — Comparisons of health-promoting lifestyle profiles (HPLP-II) between the priests and the controls. HPLP-II is a 52-item questionnaire composed of two main categories and six sub-dimension scales. The health-promoting behaviors category includes health responsibility, physical activity, and nutrition subscales. The psychosocial well-being category includes spiritual growth, interpersonal relationship, and stress management subscales. Dots represent subjects (●, priests n = 10; □, controls n = 10), and line represents median. Differences of each sub-dimension of HPLP-II were compared using a Mann–Whitney U test, indicating P value and the effect size r. (A) health responsibility (P = 0.864, r = −0.042), (B) physical activity (P = 0.837, r = −0.051), (C) nutrition (P = 0.423, r = −0.187), (D) spiritual growth (P = 0.678, r = −0.103), (E) interpersonal relationship (P = 0.615, r = −0.119), (F) stress management (P = 0.593, r = −0.127). Statistical significance was defined as P < 0.05. Cohen’s guidelines for the effect sizes (r) for Mann–Whitney U test are that a large effect is 0.5, a medium effect is 0.3, and small effect is 0.1 [28]. (TIFF 260 kb) [file 40246_2017_117_MOESM4_ESM.tif]
