# Supplementary material for: Distinct transcriptional and metabolic profiles associated with empathy in Buddhist priests: a pilot study
Source: Hum Genomics. 2017 Sep 2;11:21. doi: 10.1186/s40246-017-0117-3 (PMC5581455; doi:10.1186/s40246-017-0117-3)
Supplement: Supplementary file 5 — The empathetic process scale questionnaire. (DOCX 526 kb) [file 40246_2017_117_MOESM5_ESM.docx]

S4_Table_Ohnishi_Empathy Associated transcripts and metabolites.

| 01. I usually try to pay attention to the attitude and facial expression of other people. |
| --- |
| 02. When I see someone in distress, I feel for him or her. |
| 03. When I see people trying hard and enjoying themselves, I want to cheer for them. |
| 04. When other people are anxious, I feel anxious too. |
| 05. I often take notice of slight changes in other people’s facial expression. |
| 06. I often try to understand other’s suffering from their point of view. |
| 07. I feel sad when I see other people who are discriminated against. |
| 08. I try to put myself in somebody else’s shoes. |
| 09. When I see someone who badly needs help in a disaster, I always feel sympathetic towards him or her. |
| 10. When I see someone being emotionally moved, I often try to think of his or her point of view. |
| 11. I tend to be aware of other people’s affections and emotions. |
| 12. When I see someone being happy, I feel warm inside. |
| 13. I try to understand other people’s painful feelings by putting myself in their shoes. |
| 14. When other people are happy, I feel happy too. |
| 15. I am sensitive to someone’s mood swings. |
| 16. I feel happy when I see people enjoying themselves. |
| 17. When other people are suffering, I feel their pain. |
| 18. I compliment people who are willing to succeed. |
| 19. I think it is sad when other people are upset. |
| 20. Even if I see someone being happy, I don’t have the same feelings as that person. |
| 21. I get a warm feeling if I see other people’s joy. |
| 22. The suffering of others makes me suffer too. |
| 23. When I see someone who is willing to succeed, I feel like congratulating him or her. |
| 24. I endeavor to understand people’s feelings. |
| 25. If someone feels frightened of something, I feel frightened too. |
| 26. I feel sorry for other people when they are having problems. |
| 27. I’m able to stand in someone’s shoes and understand their happiness. |
| 28. Seeing other people happy makes me happy. |
| 29. When I am with someone who is sad, I feel his or her sadness as if it were my own. |
| 30. When I learn about someone’s good fortune, I feel happy for him or her. |
